# Hospital Versus Private Practice

**Published:** 1911-05-27

**Authors:** 


### HOSPITAL VERSUS PRIVATE PRACTICE.

The announcement has been made that in future the staff of the Johns Hopkins Hospital will be required to give up all private practice and to devote their time entirely to their clinical and teaching work in the hospital. Ever since the inauguration of this magnificently endowed and world-famous Baltimore hospital, its name has always stood for the very highest attainable standard of efficiency and progress. Is, therefore, this new departure to be taken as an indication of the future line of advance in scientific medicine and surgery?

In Britain we have no Johns Hopkins to endow medical research and educational work on the lavish scale necessary to compete successfully with Mammon and the golden opportunities of a large consulting practice. It may even be doubted whether the principle involved will find many imitators in the United States, where benefactors conduct their medical endowment schemes on such colossal lines. In Baltimore, it may be presumed, the opportunities for private practice at very high fees are much fewer and smaller than in Chicago, Boston, or New York; so that perhaps less self-denial is involved for those whom this regulation affects than at first one would be inclined to suppose. Yet even if that be so, and even if the salaries paid to the medical staff at the Johns Hopkins are sufficiently liberal, at the same time to abandon private practice altogether is to abandon material advantages of no mean order. It can only be that the desire to remain on the staff at the Johns Hopkins, and the fascinations of research work and of teaching, suffice to attract thither professors whose enthusiasm for their work is a good augury for its success.

How differently—or how much more indifferently—are things managed in Britain. Here the teachers at a clinical school are unpaid, or, at the best, wretchedly underpaid, for their labours; and they compete for posts on the staff of such schools, not from any love of teaching or from any leaning and aptitude for research, but simply from a desire for the advertisement necessary to attain a lucrative private practice. On the latter their whole energies are bent, and once they have attained it, their enthusiasm for teaching rapidly subsides; needless to say, this acts to the detriment of the ensuing generation of medical students, who will be the next generation of medical men. Surely it is as plain as anything can be that if the medical schools are starved, as everyone knows they are, the staffs of those schools must, for their own sakes and the sakes of their wives and families, make success in private practice their aim and object in life. It must be equally obvious that no blame attaches to those who do this; the system is bad, and is alone to blame. It is therefore not in the least strange that the output of men and of work from the Johns Hopkins Hospital during the comparatively short period since its foundation is, beyond dispute, far greater than that of any British medical school of the same size. Incidentally, the founders have secured for themselves, through the association of their names with the researches conducted in the hospital which they endowed, an immortality of no meagre kind. It may be one of the lowest grounds upon which to base argument or appeal for medical endowments, but since the advance of medical science is a form of human progress in which laymen can vicariously assist by providing the necessary funds, the example set to philanthropists by the Hopkins family may be emphasised at the same time as is that set to the schools of medicine by this latest innovation in their Baltimore monument.

